# Measurement-Device-Independent Quantum Key Distribution over asymmetric channel and unstable channel

**DOI:** 10.1038/s41598-018-35507-z

**Published:** 2018-12-05

**Authors:** Xiao-Long Hu, Yuan Cao, Zong-Wen Yu, Xiang-Bin Wang

**Affiliations:** 1 State Key Laboratory of Low Dimensional Quantum Physics, Tsinghua University, Beijing, 100084 People’s Republic of China; 2 National Laboratory for Physical Sciences at the Microscale and Department of Modern Physics, University of Science and Technology of China, Hefei, 230026 China; 3 CAS Center for Exellence and Synergetic Innovation Center in Quantum Information and Quantum Physics, University of Science and Technology of China, Shanghai, 201315 China; 4 Synergetic Innovation Center of Quantum Information and Quantum Physics, University of Science and Technology of China, Hefei, Anhui 230026 China; 5 Data Communication Science and Technology Research Institute, Beijing, 100191 China; 6 Shenzhen Institute for Quantum Science and Engineering, and Department of Physics, Southern University of Science and Technology, Shenzhen, 518055 China

## Abstract

We show that a high key rate of Measurement-Device-Independent Quantum Key Distribution (MDIQKD) over asymmetric and unstable quantum channel can be obtained by full optimization and compensation. Employing a gradient optimization method, we make the full optimization taking both the global optimization for the 12 independent parameters and the joint constraints for statistical fluctuations. We present a loss-compensation method by monitoring the channel loss for an unstable channel. The numerical simulation shows that the method can produce high key rate for both the asymmetric channel and the unstable channel. Compared with the existing results of independent constraints, our result here improves the key rate by 1 to tens of times in typical experimental conditions.

## Introduction

Quantum key distribution (QKD) provides the communication users with secure keys to encrypt their information. Bennett and Brassard proposed BB84 protocol^1^ to realize QKD, but the lack of practical single-photon sources limited the use of origin BB84 protocol. BB84 protocol with imperfect single-photon sources would suffer from the photon-number-splitting (PNS) attack^2–4^. This loophole can be fixed by the decoy-state method^5–8^. With the decoy-state method, QKD can be used in the practical system between users with longer distace^9–11^. After that, measurement-device-independent QKD (MDIQKD) was proposed to avoid any loophole from the imperfect detection devices^12–15^. Combined with decoy-state method, MDIQKD can also avoid the loophole from the imperfect single-photon sources^15,16^. Nowadays, the decoy-state MDIQKD has become the mainstream of the studies of quantum key distribution both theoretically^16–29^ and experimentally^30–43^. Various numerical models and optimization methods^21–24^ have improved the key rate and secure distance a lot. The numerical model by Xu *et al*.^21^ can apply to the case of asymmetrical channel rather precisely. The maximum distance of MDIQKD has been experimentally increased to 404 kilometers^38^ using the 4-intensity protocol^24^ with joint constraints for statistical fluctuations^23^. Another experiment exceeding 400-kilometer distance applying the decoy-state method but not using MDIQKD scheme was reported recently^43^.

In the scheme of decoy-state MDIQKD, at each time the user Alice (Bob) randomly chooses her (his) basis, bit value and intensity to send a pulse in a corresponding state, e.g. BB84 state in a polarization-coding MDIQKD, to an untrusted third party (UTP) Charlie. Charlie performs a collective measurement on each pulse pair and announces the measurement result in the public channel. After Charlie announces the measurement results, Alice and Bob announce the bases and intensities they use. Based on all announcement, Alice and Bob can calculate the yield and the error rate of single-photon pulse pairs, and then distill the secure key.

For the practical applications, the asymmetric and unstable channels are common cases both in fiber and free space. For example, when we consider the quantum network, due to the different geographical locations of users, the channel losses can be largely different. And if we want implement MDIQKD in free space, the channels are always asymmetric and unstable too, due to the atmospheric turbulence or moving sites (such as the satellite). Although the security of MDIQKD doesn’t make any assumption to the channel, the unstable and/or asymmetric quantum channel decreases the key rate quite a lot. Therefore, directly applying the optimized parameters for symmetric channel does not give a good performance in an asymmetric channel or an unstable channel. Here, we propose full optimization of four-intensity decoy-state MDIQKD protocol to largely increase the key rate in asymmetric channels than existing results of partial optimization. For full optimization, we mean: (1) Using 12 independent parameters, (2) Applying joint constraints^23,24^ for statistical fluctuations. If one only use one of the above two operations, that is partial optimization. Moreover, a loss-compensation method is presented with optimization to increase the performance of MDIQKD in unstable channels.

## Results

### Four-intensity decoy-state MDIQKD

Among all existing protocols of decoy-state MDIQKD, the 4-intensity protocol seems to be the most efficient one^24^ which has been extensively verified experimentally^37–40^. As was stated in the four-intensity decoy-state method in Ref.^24^, Alice and Bob each uses 4 different intensities, including one vacuum. This means in general, there are 7 different intensities for both sides with 6 independent parameters for non-vacuum intensities. Together with the frequencies of using each intensities, there are 12 independent parameters in general in the protocol. Also, the four-intensity protocol suggests using the joint constraints in statistical fluctuation of different observable^23,24^. A full implementation of the four-intensity protocol means doing optimization among all those 12 parameters with joint constraints fully.

Explicitly, in the four-intensity decoy-state method in ref.^24^, Alice (Bob) uses a source *z*_*A*_ (*z*_*B*_) with intensity *μ*_*az*_ (*μ*_*bz*_) that only emits photons in the *Z* basis, two sources *x*_*A*_ and *y*_*A*_ (*x*_*B*_ and *y*_*B*_) with intensities *μ*_*ax*_ and *μ*_*ay*_ (*μ*_*bx*_ and *μ*_*by*_) that only emit photons in the *X* basis and a vacuum source *o*_*A*_ (*o*_*B*_) that only emits vacuum pulses. At each time, Alice (Bob) randomly chooses a source in the four sources above to send a pulse, with probability ${p}_{a{l}_{A}},l=z,x,y,o\,({p}_{b{r}_{B}},r=z,x,y,o)$. So we call it “four-intensity protocol”.

The key rate of the decoy-state MDIQKD is given by:

1 $$R={p}_{az}{p}_{bz}\{{a}_{z1}{b}_{z1}\underline{\langle {s}_{11}\rangle }\,\mathrm{[1}-H\,(\overline{\langle {e}_{11}^{ph}\rangle })]-f{S}_{zz}H({E}_{zz})\}$$

where *a*_*z*1_ and *b*_*z*1_ are the fraction of single photons of sources *z*_*A*_ and *z*_*B*_, $\underline{\langle {s}_{11}\rangle }$ and $\overline{\langle {e}_{11}^{ph}\rangle }$ are the bound of yield and phase-flip error rate of single-photon pulse pairs which can be obtained by the decoy-state method, *S*_*zz*_ and *E*_*zz*_ are yield and bit-flip error rate when Alice and Bob both send pulses with source *z*_*A*_/*z*_*B*_, $H(p)=-\,p{\mathrm{log}}_{2}p-\mathrm{(1}-p){\mathrm{log}}_{2}\mathrm{(1}-p)$ is the binary entropy function and *f* is the correction efficiency.

Details for calculation of the key rate can be found in the appendix.

### Optimization of source parameters

In the numerical simulation, we will estimate what values we would observe for the yields and error rates in a certain model and use these values to calculate the key rate. Given the properties of the channel and the detection devices, we can regard the key rate as a function of source parameters:

2 $$\begin{array}{r}\tilde{R}=\tilde{R}({\mu }_{ax},{\mu }_{ay},{\mu }_{az},{p}_{ax},{p}_{ay},{p}_{az},{\mu }_{bx},{\mu }_{by},{\mu }_{bz},{p}_{bx},{p}_{by},{p}_{bz})=\tilde{R}(\overrightarrow{x}\mathrm{).}\end{array}$$

If we use weak coherent state sources, the relation between the intensity *μ* and the photon number distribution $\rho ={\sum }_{k}\,{a}_{k}|k\rangle \langle k|$ is ${a}_{k}={e}^{-\mu }\frac{{\mu }^{k}}{k!}$.

In the calculation of the key rate, we need to take the joint fluctuation and the scan of ${\mathcal H}$ (the definition of ${\mathcal H}$ is in the appendix) into consideration. In addition, the number of parameters we need to optimize is large, which means the parameter space is very huge. Therefore, normal optimization method costs a lot of time. We should improve the optimization method to get the optimal parameters quickly. Firstly, we consider the “gradient” of the key rate function^29^:

3 $$\frac{{\rm{\Delta }}\tilde{R}}{{\rm{\Delta }}{x}_{k}}=\frac{\tilde{R}({x}_{k}+{\rm{\Delta }}{x}_{k},{x}_{i})-\tilde{R}({x}_{k}-{\rm{\Delta }}{x}_{k},{x}_{i})}{2{\rm{\Delta }}{x}_{k}}.$$

In the case that both $\tilde{R}$(*x*_*k*_ + Δ*x*_*k*_, *x*_*i*_) and $\tilde{R}$(*x*_*k*_ − Δ*x*_*k*_, *x*_*i*_) are less than $\tilde{R}$(*x*_*k*_, *x*_*i*_), we set $\frac{{\rm{\Delta }}\tilde{R}}{{\rm{\Delta }}{x}_{k}}=0$. With

4 $$\frac{{\rm{\Delta }}\tilde{R}}{{\rm{\Delta }}\overrightarrow{x}}=(\frac{{\rm{\Delta }}\tilde{R}}{{\rm{\Delta }}{x}_{1}},\ldots ,\frac{{\rm{\Delta }}\tilde{R}}{{\rm{\Delta }}{x}_{12}})$$

we can find the direction that key rate increases the fastest and get close to the optimal parameters quickly.

To avoid the case that the optimal parameters are the local optimal point, which satisfies $\tilde{R}$(*x*_*k*_ + Δ*x*_*k*_, *x*_*i*_) ≤ $\tilde{R}$(*x*_*k*_, *x*_*i*_) for any *k* but is not the maximum point in the whole area, we search the points in the nearby area to see whether there is higher key rate. Accurately, we calculate the key rate $\tilde{R}$(*x*_*k*_ + *δ*_*k*_Δ*l*); *δ*_*k*_ = −1, 0, 1; *k* = 1, …, 12 with a certain Δ*l*. If there are some points with higher key rate, we jump to the point with highest key rate in the nearby area and execute the above procedure again.

In our simulation, we found that in most cases, the gradient method brings us to the optimal point. But in some cases, it brings us to the local optimal point.

### Loss-compensation method

For the case of unstable channel, according to our MDIQKD protocol, all source parameters should be determined before the QKD process and fixed during the QKD process. Even though we can detect the channel transmittance *η* at any time, we cannot change the source parameters to optimize the key rate at real time. The decoy state method requests that the intensities of pulses must be fixed. Say, switching among 3 fixed intensities. If one change the intensities beyond these 3 intensities, or change the intensities non-randomly, the result of the decoy-state method will be invalid. With the fixed source parameters *μ*_*A*_ and *μ*_*B*_ and unstable channel transmittance *η*_*A*_(*t*) and *η*_*B*_(*t*), which means the transmittance changes dependently on time, there are always some cases that the intensities at the two sides of Charlie’s beam splitter deviate a lot, saying that at some time *t*_*i*_, *μ*_*A*_*η*_*A*_(*t*_*i*_) and *μ*_*B*_*η*_*B*_(*t*_*i*_) deviate a lot. These cases will give a quite high error rate that decreases the key rate a lot.

First we consider the case with stable channel that satisfies *μ*_*A*_*η*_*A*_ > *μ*_*B*_*η*_*B*_. In an asymmetric channel, if one attenuates one path, the channel will become symmetric and the detected error rate will be small. However, in such a case, the amount of detected bits is also decreased and the final key rate is not necessarily improved. If we add extra loss $\frac{{\mu }_{B}{\eta }_{B}}{{\mu }_{A}{\eta }_{A}}\le {\tilde{\eta }}_{A}\le 1$ to the channel between Alice and Charlie, we can get a better key rate. Given the transmittance and the intensities of sources, the specific value of ${\tilde{\eta }}_{A}$ can be determined by numerical simulation.

Then we come to the case with unstable channel. Technically, the channel loss cannot be changed too fast. Otherwise the physical compensation cannot be made instantaneously. We assume that the transmittance remains unchanged during each time window, which means the transmittance doesn’t fluctuate too rapidly. Suppose that we are given the transmittance distribution $\{{\eta }_{A}^{\mathrm{(1)}},\cdots ,{\eta }_{A}^{(i)},\cdots \}$ and $\{{\eta }_{B}^{\mathrm{(1)}},\cdots ,{\eta }_{B}^{(i)},\cdots \}$ and fixed *μ*_*A*_, *μ*_*B*_. A general loss-compensation method is to add different extra loss ${\tilde{\eta }}^{(ij)}$ < 1 to Alice’s or Bob’s channel for different transmittance pair ${\eta }_{A}^{(i)}\otimes {\eta }_{B}^{(j)}$ and we can determine the optimal value of each ${\tilde{\eta }}^{(ij)}$ for each ${\eta }_{A}^{(i)}\otimes {\eta }_{B}^{(j)}$ pair. But in practice, the optimization of so many variables is out of any computer’s ability. So we keep only one extra loss $\tilde{\eta }$ and raise a variable *δ*. When the transmittance pair ${\eta }_{A}^{(i)}\otimes {\eta }_{B}^{(j)}$ satisfies ${\eta }_{A}^{(i)}/{\eta }_{B}^{(j)} > \delta$, Charlie should add the extra loss $\tilde{\eta }$ to the channel between Alice and Charlie. When the transmittance pair ${\eta }_{A}^{(i)}\otimes {\eta }_{B}^{(j)}$ satisfies ${\eta }_{B}^{(j)}/{\eta }_{A}^{(i)} > \delta$, Charlie should add the extra loss $\tilde{\eta }$ to the channel between Bob and Charlie. In other cases, Charlie doesn’t have to add any extra loss to the channel. We can use numerical simulation, combined with optimization of source parameters, to determine the values of $\tilde{\eta }$ and *δ* to get the best key rate when given a specific transmittance distribution.

In the simulation of the unstable channel, suppose that we have the transmittance distribution $\{{\eta }_{A}^{\mathrm{(1)}},\cdots ,{\eta }_{A}^{(i)},\cdots \}$, $\{{\eta }_{B}^{\mathrm{(1)}},\cdots ,{\eta }_{B}^{(j)},\cdots \}$ and the corresponding probability $\{{p}_{A}^{\mathrm{(1)}},\cdots ,{p}_{A}^{(i)},\cdots \}$, $\{{p}_{B}^{\mathrm{(1)}},\cdots ,{p}_{B}^{(j)},\cdots \}$. We can calculate the “transmittance pair distribution” $\{{\eta }_{A}^{\mathrm{(1)}}\otimes {\eta }_{B}^{\mathrm{(1)}},\cdots ,{\eta }_{A}^{(i)}\otimes {\eta }_{B}^{(j)},\cdots \}$ and the corresponding probability $\{{p}_{A}^{\mathrm{(1)}}\ast {p}_{B}^{\mathrm{(1)}},\cdots ,{p}_{A}^{(i)}\ast {p}_{B}^{(j)},\cdots \}$. With a certain source pair *lr* and a certain transmittance pair ${\eta }_{A}^{(i)}\otimes {\eta }_{B}^{(j)}$, the observed yield ${S}_{lr}({\eta }_{A}^{(i)}\otimes {\eta }_{B}^{(j)})$ and the observed error rate ${E}_{lr}({\eta }_{A}^{(i)}\otimes {\eta }_{B}^{(j)})$ can be calculated as in ref.^44^ theoretically. Then the yield and the error rate in the whole process can be calculated by

5 $${S}_{lr}=\sum _{i,j}\,{p}_{A}^{(i)}{p}_{B}^{(j)}{S}_{lr}({\eta }_{A}^{(i)}\otimes {\eta }_{B}^{(j)})$$

and

6 $${E}_{lr}=\sum _{i,j}\,{p}_{A}^{(i)}{p}_{B}^{(j)}{E}_{lr}({\eta }_{A}^{(i)}\otimes {\eta }_{B}^{(j)}\mathrm{).}$$

When the loss-compensation is performed, we can calculate the *S*_*lr*_, *E*_*lr*_ in the same way except the (*ij*)-th transmittance pair is changed into ${\eta }_{A}^{(i)}\tilde{\eta }\otimes {\eta }_{B}^{(j)}$ if $\frac{{\eta }_{A}^{(i)}}{{\eta }_{B}^{(j)}} > \delta$, or ${\eta }_{A}^{(i)}\otimes {\eta }_{B}^{(j)}\tilde{\eta }$ if $\frac{{\eta }_{B}^{(j)}}{{\eta }_{A}^{(i)}} > \delta$.

### Numerical simulation

**Figure 1:**
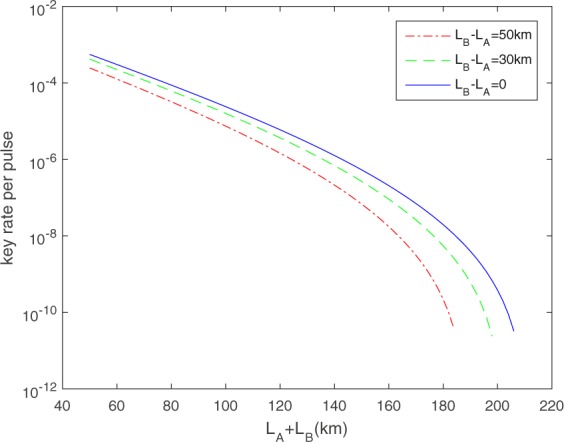
Optimized key rate versus the total distance between Alice and Bob in asymmetric channel with the device parameters in Table 2.

First we consider the case that the channel is stable but asymmetric. We shall estimate what values would be probably observed for the yields in the normal cases by the linear models^15,21,22^ and the errors in X basis by the useful model for misalignment error under asymmetric channel in ref.^21^. We show the optimized key rate in some asymmetric cases in Fig. 1 and some results in certain distances in Table 1, in comparison with the results of independent bounds in ref.^45^, with device parameters in Table 2. From Fig. 1, we can find that with full implementation of the four-intensity MDIQKD and full optimization of the source parameters, quite good key rate can be achieved. In particular, we have taken the joint constraints^23,24^ for statistical fluctuations, this affects the key rate significantly. Taking joint constraints will consume some more computational time. The computation can be done very fast if we apply formulas of refs^23,24^ rather than the linear programming. In our numerical simulation, we have already overcome the problem of unpredictable behavior such as jitters^45^. Actually, the computational time seems not to be a major issue for the practical application. In the application for the unstable channel, one can choose to test the channels and do the optimization for the major loss values of the changing channel before running the protocol.

**Table 1:** Optimized key rate at different distances in asymmetric channel with the parameters in Table 2.

*L*_*A*_(km)	*L*_*B*_(km)	Optimized key rate per pulse pair	
		ours	Ref.^45^
10	60	6.299 × 10^−5^	3.106 × 10^−5^
43	93	3.151 × 10^−7^	1 × 10^−8^
50	100	6.576 × 10^−8^	4.786 × 10^−11^
30	60	3.117 × 10^−5^	1.445 × 10^−5^
59.3	89.3	2.972 × 10^−7^	1 × 10^−8^
70	100	2.490 × 10^−8^	0

**Table 2:** Device parameters for Table 1.

*N*_*t*_	*η*_*d*_	*d*	${{\boldsymbol{E}}}_{{\boldsymbol{d}}}^{{\boldsymbol{X}}}$	${{\boldsymbol{E}}}_{{\boldsymbol{d}}}^{{\boldsymbol{Z}}}$	*f*	*ε*
10^11^	65%	8 × 10^−7^	0.5%	0.5%	1.16	10^−7^

*N*_*t*_: total number of pulse pairs; *η*_*d*_: detection efficiency of the detectors; *d*: dark count rate of the detectors; ${E}_{d}^{X}$/${E}_{d}^{Z}$: misalignment error rate in the *X*/*Z* basis; *f*: correction efficiency*; ε*: failure probability for statistical fluctuation evaluation between observable and the mean value.

Then for the unstable channel, we consider a simple case that ${\eta }_{A}^{(i)}=\mathrm{(3}+2i)$ dB, ${\eta }_{B}^{(j)}=\mathrm{(13}+2j)$ dB with probability ${p}_{A}^{(i)}={p}_{B}^{(j)}=0.2$ for *i*, *j* = 1, …, 5 (Here the detector’s efficiency is contained in the total loss). We show the key rate with different *δ* and $\tilde{\eta }$ with source parameters optimized in Table 3.

**Table 3:** Optimized key rate with different *δ* and $\tilde{\eta }^{\prime}$ in unstable channel with the parameters in Table 2.

*δ*(dB)	$\tilde{\eta }^{\prime}$(dB)	Optimized key rate per pulse pair
0	0	1.7747 × 10^−6^
−7	5	1.5229 × 10^−6^
−8.5	4.5	2.2279 × 10^−6^
−8.75	4	2.2217 × 10^−6^
−8.75	4.5	2.2283 × 10^−6^
−8.75	5	2.2184 × 10^−6^
−9	4.5	2.2278 × 10^−6^

The case that *δ* = $\tilde{\eta }^{\prime}$ = 0 dB is equal to that we don’t perform the loss-compensation method. In the second line of Table 3, we can see that an improper loss-compensation (*δ* = −7 dB and $\tilde{\eta }^{\prime}$ = 5 dB) will decrease the key rate. We can find that in this transmittance distribution, setting *δ* = −8.75 dB and $\tilde{\eta }^{\prime}$ = 4.5 dB can maximize the key rate in our loss-compensation method.

## Discussion

We propose a full implementation of four-intensity decoy-state MDIQKD. Even if the channels between the users and UTP are asymmetric, our four-intensity protocol still has a good performance. We also propose a loss-compensation method. This method can improve the key rate a lot in unstable channel.

### Method: calculation of the key rate

#### Asymptotic case

We define the yield, the error yield and the error rate as follow. Consider a pulse pair set ${\mathscr{C}}$, which contains ${N}_{{\mathscr{C}}}$ pulse pairs totally. These pairs cause ${M}_{{\mathscr{C}}}$ effective counts and ${W}_{{\mathscr{C}}}$ error counts. In this case, the yield ${S}_{{\mathscr{C}}}={M}_{{\mathscr{C}}}/{N}_{{\mathscr{C}}}$, the error yield ${T}_{{\mathscr{C}}}={W}_{{\mathscr{C}}}/{N}_{{\mathscr{C}}}$ and the error rate ${E}_{{\mathscr{C}}}={W}_{{\mathscr{C}}}/{M}_{{\mathscr{C}}}$.

In photon number space, the density matrices of the pulses from the sources can be written as

7 $${\rho }_{{l}_{A}}=\sum _{k=0}^{\infty }\,{a}_{lk}|k\rangle \langle k|,\,l=x,y,z$$

and

8 $${\rho }_{{r}_{B}}=\sum _{k=0}^{\infty }\,{b}_{rk}|k\rangle \langle k|,\,r=x,y,z.$$

We assume that the states above satisfy these conditions:

9 $$\frac{{a}_{yk}}{{a}_{xk}}\ge \frac{{a}_{y2}}{{a}_{x2}}\ge \frac{{a}_{y1}}{{a}_{x1}},\,\frac{{b}_{yk}}{{b}_{xk}}\ge \frac{{b}_{y2}}{{b}_{x2}}\ge \frac{{b}_{y1}}{{b}_{x1}}$$

for *k* > 2, so that the decoy-state results can apply. Familiar sources used in practice, such as weak-coherent-state sources and heralded single-photon sources out of the parametric-down conversion, satisfy the conditions above.

In the following, we will omit the subscript *A* and *B* in *l*_*A*_ and *r*_*B*_ if it doesn’t cause confusion. A pulse pair of *lr* is a pair where Alice’s pulse is from *l* and Bob’s pulse if from *r*. The two-mode source *lr* emits all *lr* pairs.

The main idea of decoy state is that the yield of |*m*〉|*n*〉 photon pairs from different source pairs should be the same in the asymptotic case, which means

10 $$\langle {s}_{mn}^{lr}\rangle =\langle {s}_{mn}\rangle ,\,l,r=x,y,z.$$

Using Eq. (10) and the convex form of yield of *lr* source pairs

11 $$\langle {S}_{lr}\rangle =\sum _{m,n=0}^{\infty }\,{a}_{lm}{b}_{rn}\langle {s}_{mn}\rangle ,$$

we can calculate the lower bound of the yield of single-photon pairs:

12 $$\langle {s}_{11}\rangle \ge \underline{\langle {s}_{11}\rangle }=\frac{{S}_{+}-{S}_{-}-{a}_{y1}{b}_{y2} {\mathcal H} }{{a}_{x1}{a}_{y1}({b}_{x1}{b}_{y2}-{b}_{x2}{b}_{y1})}$$

where

13 $${S}_{+}={a}_{y1}{b}_{y2}\langle {S}_{xx}\rangle +{a}_{x1}{b}_{x2}{a}_{y0}\langle {S}_{oy}\rangle +{a}_{x1}{b}_{x2}{b}_{y0}\langle {S}_{yo}\rangle ,$$

14 $${S}_{-}={a}_{x1}{b}_{x2}\langle {S}_{yy}\rangle +{a}_{x1}{b}_{x2}{a}_{y0}{b}_{y0}\langle {S}_{oo}\rangle $$

and

15 $$ {\mathcal H} ={a}_{x0}\langle {S}_{ox}\rangle +{b}_{x0}\langle {S}_{xo}\rangle -{a}_{x0}{b}_{x0}\langle {S}_{oo}\rangle \mathrm{.}$$

Eq. (12) holds when

16 $${K}_{a}=\frac{{a}_{y1}{a}_{x2}}{{a}_{x1}{a}_{y2}}\le \frac{{b}_{y1}{b}_{x2}}{{b}_{x1}{b}_{y2}}={K}_{b}.$$

In the case of *K*_*a*_ > *K*_*b*_, the lower bound of *s*_11_ can be calculated with Eqs (12–15) by making exchange between *a*_*xk*_ and *b*_*xk*_, and exchange between *a*_*yk*_ and *b*_*yk*_, for *k* = 1, 2.

Similarly, we can calculate the upper bound of phase-flip error rate of single-photon pairs:

17 $$\langle {e}_{11}^{ph}\rangle \le \overline{\langle {e}_{11}^{ph}\rangle }=\frac{\langle {T}_{xx}\rangle - {\mathcal H} \mathrm{/2}}{{a}_{x1}{b}_{x1}\underline{\langle {s}_{11}\rangle }}.$$

With $\underline{\langle {s}_{11}\rangle }$ and $\overline{\langle {e}_{11}^{ph}\rangle }$, we can calculate the key rate with Eq. (1).

### Nonasymptotic case

In the nonasymptotic regime, we should consider the statistical fluctuation of the observable, e.g. the difference between observed values and mean values. Given a failure probability *ε*, the observed value ${S}_{{\mathscr{C}}}$ of an observable of a set ${\mathscr{C}}$ and its mean value $\langle {S}_{{\mathscr{C}}}\rangle$ satisfy:

18 $$-{{\rm{\Delta }}}_{-}\le {S}_{{\mathscr{C}}}-\langle {S}_{{\mathscr{C}}}\rangle \le {{\rm{\Delta }}}_{+}.$$

If we perform a standard error analysis, Δ can be given by

19 $${{\rm{\Delta }}}_{-}={{\rm{\Delta }}}_{+}=\gamma \sqrt{\frac{{S}_{{\mathscr{C}}}}{{N}_{{\mathscr{C}}}}}$$

where ${N}_{{\mathscr{C}}}$ is the number of elements in set ${\mathscr{C}}$ and *γ* = 5.3 given the failure probability *ε* = 10^−7^.

According to the idea of joint constraints of statistical fluctuation^23^, the set ${\mathscr{C}}$ can be either all pulse pairs from a source pair, or the combination of all pulse pairs from different source pairs. Taking all these joint constraints into consideration, the bound of 〈*s*_11_〉 and $\langle {e}_{11}^{ph}\rangle$ can be calculated tighter.

As a joint term in $\underline{\langle {s}_{11}\rangle }$ and $\overline{\langle {e}_{11}^{ph}\rangle }$, ${\mathcal H}$ should fluctuate jointly in Eq. (12) and Eq. (17), instead of taking the worst case independently^24^. We regard *R* as a function of ${\mathcal H}$, scan ${\mathcal H}$ in its possible range and take the minimum *R* as the final key rate:

20 $$\tilde{R}=\mathop{min}\limits_{ {\mathcal H} \in [\underline{ {\mathcal H} },\overline{ {\mathcal H} }]}R( {\mathcal H} )$$

### Method: verifying the global optimization

When we search the points in the nearby area in our optimization algorithm, we can change the parameter Δ*l* in our programme. In the cases with different Δ*l*, e.g. Δ*l* = 0.1, 0.001, 0.0001…, we obtain almost the same optimized results.

We make another test that to each point, we start from many sets of different initial values of parameters. We then obtain almost the same results for the optimized parameters.

These strongly indicate that our result is indeed the globally optimized result.
